# Glia maturation factor gamma regulates the migration and adherence of human T lymphocytes

**DOI:** 10.1186/1471-2172-13-21

**Published:** 2012-04-18

**Authors:** Dustin ND Lippert, John A Wilkins

**Affiliations:** 1 Manitoba Centre for Proteomics and Systems Biology, University of Manitoba, Winnipeg, Canada; 2 Departments of Internal Medicine and Biochemistry and Medical Genetics, University of Manitoba, Winnipeg, Canada

**Keywords:** T lymphocytes, Chemotaxis, CXCL12, Pseudopodia, Glia maturation factor gamma, GMFG, Proteomics, ShRNAmir, Adhesion

## Abstract

**Background:**

Lymphocyte migration and chemotaxis are essential for effective immune surveillance. A critical aspect of migration is cell polarization and the extension of pseudopodia in the direction of movement. However, our knowledge of the underlying molecular mechanisms responsible for these events is incomplete. Proteomic analysis of the isolated leading edges of CXCL12 stimulated human T cell lines was used to identify glia maturation factor gamma (GMFG) as a component of the pseudopodia. This protein is predominantly expressed in hematopoietic cells and it has been shown to regulate cytoskeletal branching. The present studies were undertaken to examine the role of GMFG in lymphocyte migration.

**Results:**

Microscopic analysis of migrating T-cells demonstrated that GMFG was distributed along the axis of movement with enrichment in the leading edge and behind the nucleus of these cells. Inhibition of GMFG expression in T cell lines and IL-2 dependent human peripheral blood T cells with shRNAmir reduced cellular basal and chemokine induced migration responses. The failure of the cells with reduced GMFG to migrate was associated with an apparent inability to detach from the substrates that they were moving on. It was also noted that these cells had an increased adherence to extracellular matrix proteins such as fibronectin. These changes in adherence were associated with altered patterns of β1 integrin expression and increased levels of activated integrins as detected with the activation specific antibody HUTS4. GMFG loss was also shown to increase the expression of the β2 integrin LFA-1 and to increase the adhesion of these cells to ICAM-1.

**Conclusions:**

The present studies demonstrate that GMFG is a component of human T cell pseudopodia required for migration. The reduction in migration and increased adherence properties associated with inhibition of GMFG expression suggest that GMFG activity influences the regulation of integrin mediated adhesion.

## Background

T Lymphocytes are involved in various aspects of immune functions such as surveillance, inflammation and wound healing. These activities are dependent upon the chemotaxis of immune cells to sites of antigen deposition or inflammation [1,2]. Lymphocyte chemotaxis involves an iterative series of coordinated molecular events including cell polarization, adhesion and force generation [3,4]. The molecular processes required for cell movement and recruitment are not fully understood. This derives in part from an incomplete knowledge of the cellular components that are required for chemotaxis. We performed a detailed mass spectrometry based compositional analysis of the isolated pseudopodia of migrating T lymphocytes as an approach to understanding the molecular basis for these processes. One of the components identified in pseudopodia was Glia maturation factor γ (GMFG) suggesting that it might play a role in the migration or chemotaxis of T lymphocytes. While the current studies were in progress it was reported that GMFG is required for the chemotaxis of human neutrophils [5].

GMFG is a 17 kDa protein which, contrary to the name, is not involved in the development of glia or the formation of gliomas [6]. Rather it is highly expressed in the thymus, spleen, lung and in highly motile cell types such as lymphoblasts, T-lymphocytes, macrophages and fibroblasts [7,8]. GMFG has a high level of structural similarity with members of the ADF (actin depolymerisation factor) domain containing family suggesting that it might play a role in the remodeling of the actin cytoskeleton [8]. Although an early study suggested that GMFG directly interacted with actin filaments [7], subsequent reports using purified actin and a GMFG homolog from yeast have yielded conflicting results [9,10]. Several groups have shown that GMFG and its homologs interact with purified Arp2/3 complex [7,9,10] and interfere with Arp2/3 induced daughter filament growth. They also cause removal of actin branches from the parent actin filament (i.e. pruning). These observations suggested that GMFG may be involved in cell movement, possibly by defining the location of Arp2/3 dependent actin branching required for membrane extension or by providing a mechanism for the regeneration of the actin monomer pool required for continued cell pseudopodia extension.

The present study demonstrates that GMFG is required for the migration and chemotaxis of human T-lymphocytes. The loss of GMFG results in reduced cell migration which appears to be associated with altered integrin expression and increased cell adhesion. These results suggest that GMFG may be required for the proper control of cellular adhesion which is essential for cell movement.

## Results

### GMFG is present in the pseudopodia of SDF1 treated T-lymphocytes

The intent of our studies was to examine the roles of proteins found in the pseudopodia of chemotactically stimulated lymphocytes. Pseudopodia of SDF1 stimulated Jurkat cells were isolated from the undersides of transwell insert membranes containing 3 μm pores. The pores prevented full transmigration but did permit pseudopodia extensions through the membrane pores. The proteins in the pseudopodia were subsequently identified by mass spectrometry.

**Figure 1:**
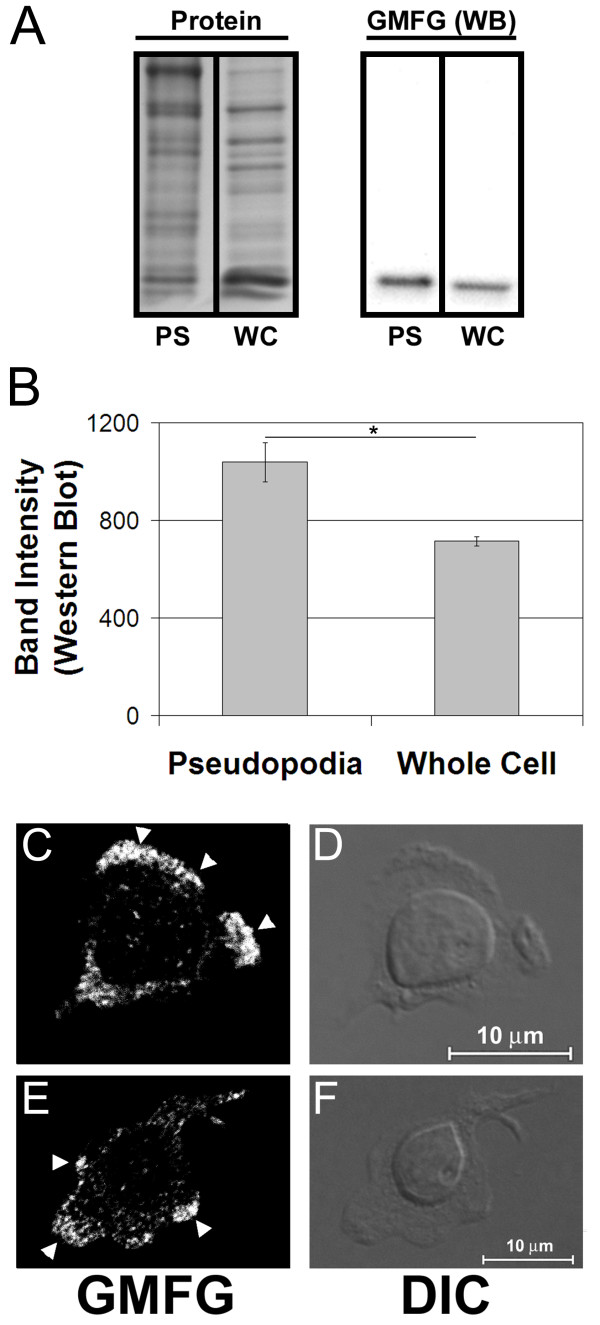
**GMFG is enriched in lymphocyte pseudopodia. Panel A)** The proteins in isolated pseudopodia (PS) or whole cell lysates (WC) of Jurkat T cells separated by SDS-PAGE. The levels and protein patterns were determined by staining for total protein (left panels) and the levels of GMFG in each fraction were assessed by western blot (right panels). **Panel B**) Quantitation of the intensity of the GMFG bands observed in the western blots in A. The GMFG levels were 45% greater in the pseudopodia than in the whole cell lysate (n = 3, * *p* = 0.017). **Panels C-F)** Confocal images of GMFG distribution in IL-2 dependent T cells. **C, E)** Arrowheads indicate the presence of GMFG at the leading edge of the cell. **D, F)** Difference interference contrast images of the same cells for comparison of cell morphology.

Glia maturation factor gamma was consistently identified by mass spectrometry in all of the pseudopodia isolates from SDF1 stimulated Jurkat. The peptides that were identified for GMFG are listed in Additional file 1. However, GMFG was not detected when the same amount of protein from whole cell lysates was examined by mass spectrometry under identical conditions. Clearly GMFG must be present in the lysates of cells that the pseudopodia were isolated from. However, the presence of GMFG was potentially masked by the presence of higher abundance proteins that were reduced in the pseudopodia preparations. A western blot based comparison of GMFG levels in pseudopodia and cell lysates, confirmed that GMFG was enriched ~1.5-fold in pseudopodia relative to whole cell lysates (Figure 1A&B). Immunofluorescence microscopy further demonstrated that GMFG was recruited to the leading edge of SDF1 stimulated IL-2 dependent peripheral blood T-lymphocytes (arrowheads, Figure 1C&E). However, GMFG was also detected throughout the cell body particularly in the region immediately behind the nuclei of migrating cells. Similar distributions were also observed in migrating Jurkat and CCRF-CEM cells (not shown). These results indicated that a component of the total cellular GMFG pool was recruited to the leading edges of migrating T cells and enriched relative to the cell bodies.

### GMFG is required for SDF1 induced T-lymphocyte chemotaxis

**Figure 2:**
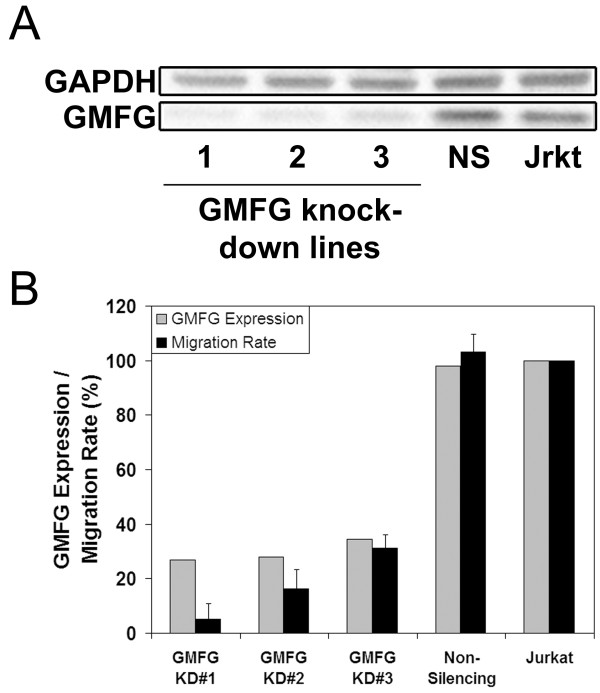
**Effects of shRNA mediated reduction of GMFG expression on T cell migration. A)** The efficiency of GMFG knockdown in Jurkat cells was assessed by western blot. Lanes 1–3 contained lysates from lines generated with three different shRNA targeting GMFG. Lanes NS and Jrkt respectively contained cells transduced with a non-silencing control vector or untreated Jurkat cells. **B)** The levels of GMFG expression (grey bars) and chemotactic responses (black bars) of each cell line were compared. Values are presented as a percentage of the wild type expression and migration levels (N = 3, +/− SEM).

The accumulation of GMFG in the leading edge of the cell suggested that it could be involved in cell migration. The role of GMFG in these processes was examined using shRNA to reduce GMFG protein expression levels. Jurkat cells were transduced with one of three distinct shRNA clones that targeted different regions of the GMFG sequence. Western blot analysis of GMFG levels demonstrated that each of these clones caused a 65–76% reduction in GMFG levels relative to cells transduced with a non-silencing control vector (Figure 2A). The control transductants had GMFG levels similar to those of wild type Jurkat cells suggesting that the transduction process per se did not impact on the expression of this protein.

Reduction of GMFG expression levels did not impact on cellular architecture or proliferation suggesting that it was not essential for these processes. However, the SDF1 induced chemotaxis of cells with reduced GMFG was inhibited by 69–95% compared with either non-silencing control transductants or untransduced Jurkat cells (Figure 2B).

**Figure 3:**
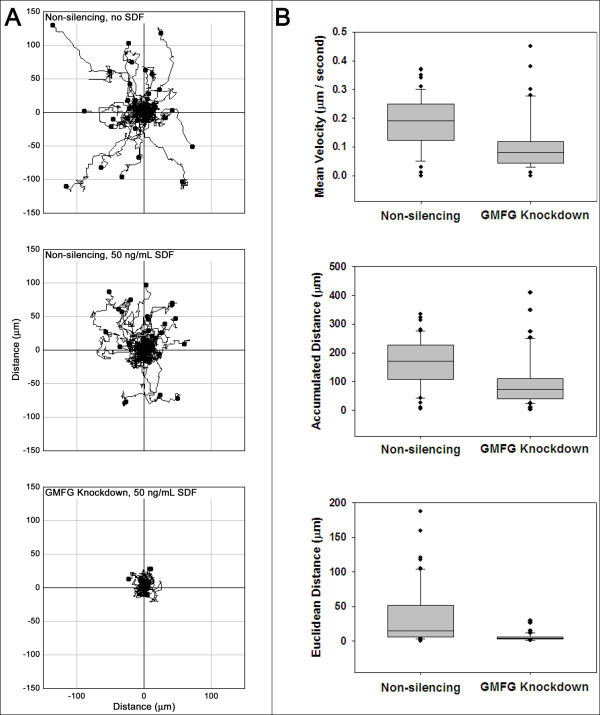
**The effects of GMFG knockdown on T cell movement. Panel A)** Control IL-2 dependent T cells containing a non-silencing construct displayed random movement on a fibronectin coated surface (upper panel) which was not enhanced by the addition of SDF1 (middle panel). GMFG knockdown resulted in the abrogation of most of the detectable movement, even in the presence of SDF1 (lower panel). **Panel B)**. Significant reductions were observed for the velocity (upper panel), accumulated distance (middle panel) and Euclidean distance (lower panel) of GMFG knockdown cells relative to control cells. The difference between non-silencing and GMFG knockdown groups was statistically significant ( *p* < 0.001) in all three parameters.

The loss of chemotaxis could arise from changes in cellular motility or a failure of cells to respond to chemokine stimulation. The effects of GMFG knockdown (GMFG-KD) on migration rates were examined in IL-2 dependent T cells. Cell tracking plots clearly demonstrated the reduced motility of GMFG-KD cells in comparison to non-silencing controls (Figure 3A). A closer inspection of the knockdown cells showed that they were able to polarize and extend pseudopodia. However, the knockdown cells were unable to fully detach from their initial positions and the observed pseudopodia were rapidly collapsed and formed at one or more new sites (see Additional file 2, Additional file 3, Additional file 4, Additional file 5 and Additional file 6). This process was repeated continuously, with little or no net movement observed for the knockdown cells. Quantitation of tracking data comparing knockdown cells to controls indicated a 58% decrease in median velocity, a 57% decrease in median accumulated distance and a 74% decrease in the median Euclidean distance traveled (Figure 3B). Thus, the apparent accumulated distance traveled by these cells, was actually a measure of repeated extension and retraction of cellular processes from a fixed point. In reality there was no net movement. These observations raised the possibility that GMFG played a role in the regulation of cellular adherence.

### The effects of GMFG loss on T cell adhesion and integrin expression

**Figure 4:**
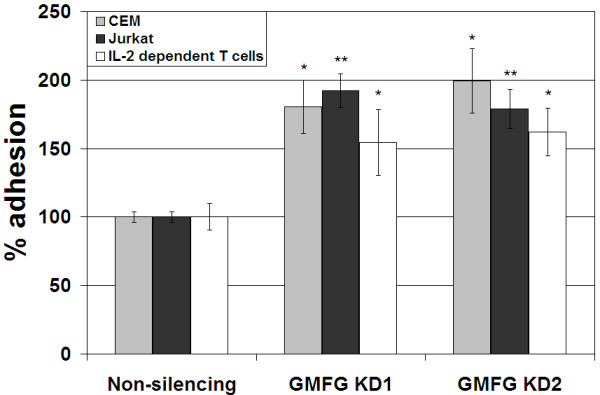
**The effects of GMFG knockdown on T cell adherence.** The impact of GMFG knockdown on Jurkat, CCRF-CEM, and IL-2 dependent T cell adherence to immobilized fibronectin was compared relative to that of their corresponding control cells. Two different GMFG targeting shRNAs were used to generate distinct cell lines (GMFG KD1 and GMFG KD2). Adhesion is expressed as a percentage of the adherence of the corresponding non-silencing control cells (N = 3, +/− SEM; * *p* < 0.05; ** *p* < 0.005).

The relative levels of adhesion of GMFG-KD cells to fibronectin was 1.5 to 2.0 fold that of nonsilencing control cells (Figure 4). The increased adherence was observed in Jurkat cells, CCRF-CEM cells and IL-2 dependent peripheral blood T-lymphocytes using two different GMFG shRNA constructs for each cell line.

**Figure 5:**
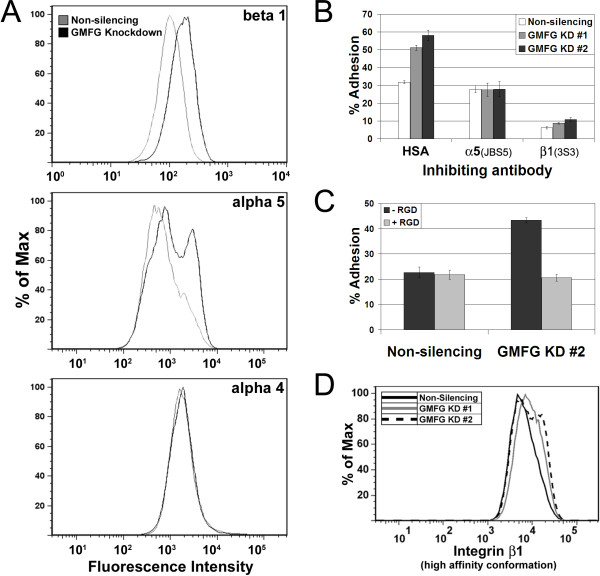
**(A) The effects of GMFG knockdown on CCRF-CEM T cell integrin expression levels and functional activities. A)** The levels of β1, α4 and α5 integrin expression were determined by flow cytometry for a non-silencing control and GMFG knockdown cells (representative example of 3 experiments). **B)** The effects of a control (HSA), or adhesion blocking antibodies to integrin on the adhesion of control and GMFG knockdown cells to fibronectin. **C)** The effects of an RGD containing peptide on the adhesion of non-silencing and GMFG knockdown cells to fibronectin. In the absence of peptide, the number of adherent GMFG knockdown cells was 91% higher than control cells ( *p* < 0.001). The RGD peptide did not have any significant effect on the adherence of non-silencing control cells. Data is shown for cell lines created from CCRF-CEM T cells and is representative of two separate experiments. **D)** The effects of GMFG knockdown on the expression levels of the high-affinity binding conformation of β1 integrin. The control and GMFG-KD cells were stained with HUTS-1. HUTS-1 staining was greater in knockdown cells (grey and dotted lines) than in comparable non-silencing control cells (black line). Data is representative of two separate experiments.

Members of the β1 integrin family are the predominant mediators of lymphocyte cell adhesion to extracellular components such as fibronectin [11]. The surface expression of β1 integrin on GMFG-KD cells was increased by 61% compared with control cells (Figure 5A). Both α4β1 and α5β1 integrins have been shown to contribute to lymphocyte fibronectin binding. There was a 66% increase in α5 integrin levels in GMFG-KD cells. In contrast, the levels of the α4 subunit were unaffected in the GMFG-KD cells. These results indicated that the loss of GMFG was associated with altered β1and α5 expression levels and patterns.

The contributions of integrins to the adhesive changes were examined using adhesion blocking antibodies specific for the β1 (3S3) and α5 (JBS5) subunits. Treatment with 3S3 (reduced the adhesion of all three cell lines to less than 10% of the input cells suggesting that β1 integrins are responsible for the bulk of the adhesion of these cells to fibronectin (Figure 5B). In contrast JBS5 appeared to only inhibit the increased level of adhesion found in the GMFG-KD cells implying that the elevated adhesion of these cells to fibronectin was mediated by α5β1.

The α5β1 integrin binds specifically to an RGD motif within fibronectin [12,13] and this interaction can be inhibited with synthetic peptides containing the RGD sequence. The addition of RGD peptide to cell suspensions of a CCRF-CEM GMFG-KD line reduced the fibronectin adhesion of these cells to levels comparable to those of control non-silencing cells (Figure 5C). Similar results were obtained for a Jurkat GMFG-KD line (not shown). Interestingly, RGD had no discernible effect on the binding of control cells to fibronectin. These data further support the conclusion that the increased adhesion of GMFG-KD cells is due to an increase in the levels of functional α5β1 integrin on the surface of these cells.

Integrins have multiple conformations corresponding to different affinities [14,15]. Changes to the activation state of α5β1 integrins could contribute to the observed increase in adhesion of the GMFG-KD lines. The HUTS-4 antibody is specific for the active conformation of β1 integrins [16]. Flow cytometry analysis of GMFG-KD lines and controls using this antibody demonstrated an increase in the amount of active β1 integrin on the surface of GMFG-KD cells (Figure 5D). These data demonstrated that increased α5β1 integrin was accompanied by an increase in the activation state of the β1 subunit suggesting that changes in the levels and activities of α5β1 are responsible for the increased adhesion of GMFG-KD cells to fibronectin.

### GMFG loss is associated with altered β1 integrin distribution

**Figure 6:**
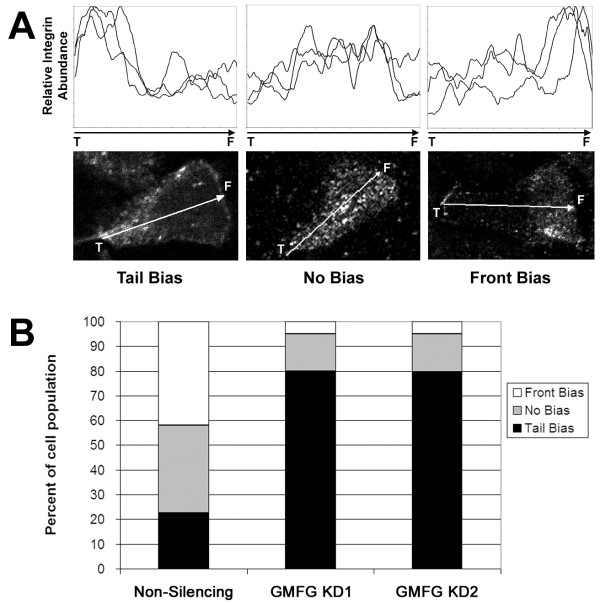
**The effects of GMFG knockdown on β1 integrin distribution in CCRF-CEM cells.** Control and GMFG-KD cells were plated on fibronectin coated surfaces, fixed and stained for β1 integrin expression. The intensity of staining fluorescence was measured along the length of the cell. Cells were categorized as having a tail bias, no bias or front bias with respect to integrin distribution. The tail and front of each cell is indicated respectively by ‘T’ and ‘F’. **A)** Line plots for three representative cells and an image of a cell in each category. **B)** The proportions of cells in each category. Results are from the analysis of 60 cells from each cell line.

The distribution of β1 integrins on non-silencing and GMFG-KD cells was examined by confocal immunofluorescent microscopy. Fluorescence intensity plots were constructed along lines drawn through the central polarized axis of T-cells (Figure 6A). The distributions of the β1 integrins along these axes were then categorized as either tail biased, unbiased or front biased. Sixty cells from each cell line were processed in this fashion as a sampling of the overall distribution of β1 integrins. Line plots from three representative cells from each category were overlaid to produce the graphs shown in Figure 6A, along with a single cell image to illustrate the staining patterns. GMFG-KD cells predominantly displayed a tail bias (80% for each knockdown line) while only 22% of control cells fell into this category (Figure 6B). The knockdown cells were rarely observed to have a front bias (~5%), while the control cells were evenly split between front biased and unbiased (~40% each).

### GMFG loss leads to increased β2 integrin expression and adhesion to ICAM-1

**Figure 7:**
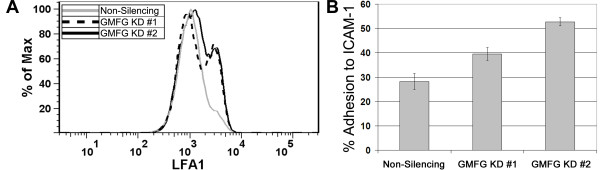
**The effects of GMFG knockdown on LFA-1 expression and function of CCRF-CEM cells. A)** GMFG knockdown cells (dotted and black lines) show an increase in the relative surface expression of LFA-1 when compared to control cells (grey line). **B)** The adhesion of GMFG knockdown cells to ICAM-1 was increased in comparison to a control non-silencing line. The difference observed was statistically significant ( *p* < 0.05) for each of the knockdown lines when compared to the control. The results shown represent the mean of three experiments +/− the standard error.

Analysis of LFA-1 (α_L_β2 integrin) surface expression on GMFG-KD cells revealed a modest but consistent increase (Figure 7A). The increase ranged from 58 to 61% above that of controls in both the knockdown lines examined. Similar results were seen with both CCRF-CEM and Jurkat cells (not shown). This increased surface expression of LFA-1 correlated with an enhanced adherence of these cells to ICAM-1 relative to non-silencing control cells (Figure 7B). The two knockdown lines tested showed statistically significant increases (*p* < 0.05) in adhesion by 43% and 85% (mean of three experiments).

## Discussion

The present study demonstrated that GMFG is enriched in the pseudopodia of migrating human T lymphocytes. Interference with GMFG expression markedly inhibited SDF1 stimulated chemotaxis. This effect was associated with reduced migration and a failure to sustain cellular polarity and directed movement. The inability of the cells to move was associated with an increase in total β1 integrin levels which included elevated expression levels of the high-affinity conformation of the β1 subunit. These changes and the increased adhesion of GMFG-KD cells to fibronectin appeared to be predominantly related to increased α5β1 levels. This was further supported by the fact that a synthetic RGD containing peptide and inhibitory antibodies specific for the α5 and β1 subunits reduced the elevated adhesion of the GMFG-KD lines to levels comparable with control cells. The loss of GMFG also seems to impact on the distribution of integrin molecules, as there is a propensity for GMFG-KD cells to accumulate β1 integrins within the uropod. This accumulation, in combination with increased surface expression levels, was consistent with failure of the cells to detach from the substrate. Furthermore the changes in integrin levels and activity were not restricted to the α5β1 integrin suggesting that GMFG activity plays a broader role in control of cellular adhesion.

GMFG is a member of the actin-depolymerizing factor homology (ADF-H) family with actin depolymerizing activity [7]. Recent studies indicate that GMFG mediated depolymerization differs from that of other ADF-H members such as cofilin [10]. GMFG does not bind directly to actin, rather it interacts with Arp2/3 complex members which can induce debranching of actin filaments. Interestingly, while GMFG reduces the rate of actin filament assembly, the distribution and absolute amount of F-actin ultimately produced during in vitro assays is unaltered in the absence of GMFG (data not shown). These observations raise the possibility that GMFG may be required for the transition from branched actin networks at the leading edge to more filamentous structures that are at immediately proximal to the leading edge of migrating cells. These filaments may provide the cytoskeletal architecture for interaction with integrins through integrin associated actin binding proteins. Such interactions could also regulate integrin activation and distribution to generate the adhesion required for cell movement.

Several GMFG interaction partners have been identified to date. GMFG was identified in immunoprecipitates of Arp2/3 complexes in Hela cells that were overexpressing GMFG [7]. Subsequently, several groups confirmed the interaction of murine, yeast or dictyostelium GMFs and the Arp2/3 complex using purified proteins [9,10]. WAVE2 was also co-precipitated with Myc tagged GMFG in dHL60 [5]. However it is unclear if the latter interaction relates to cell motility rather than chemotaxis as these results were obtained with cells that had not been treated with exogenous chemokine.

The present studies demonstrated a role for GMFG in the control of T cell adhesion and its subsequent effects on cell migration in stably transduced lymphocyte lines and short term IL2 dependent peripheral blood T cells. The loss of GMFG has also been shown to decrease the chemotactic responses of both PMN and differentiated HL60 cells [5]. The phosphorylation levels of p21 activated kinases (PAK 1 or PAK 2) and p38 MAPK were also found to be decreased following fMLF or CXCL8 stimulation of dHL60 cells. PAK1/2 plays multiple roles in the regional regulation of actin dynamics and myosin function [17]. The strength of focal adhesions can be regulated by Pak1/2 activity as inhibition of either the activity or expression of Pak1/2 results in increased numbers of nascent long lived adhesions. The net effect of these alterations is a reduction in cellular migration. Additionally, the conformation of α5β1 integrin is known to switch from low to high affinity in focal adhesions [18]. Hence the increased proportion of activated β1 integrin observed on GMFG-KD cells would be consistent with a Pak1/2 mediated increase in the number of focal adhesions. An intriguing possibility is that GMFG alters cytoskeletal organization which impacts on the frequency and properties of integrin contacts with the extracellular matrix. The strength of α5β1 binding can be enhanced by cytoskeletal tension forces generated by myosin II [19], and it is conceivable that GMFG could be involved in the regulation of force generation by its effects on the cytoskeleton. The observed changes in the integrin expression patterns and activity may be a reflection of architectural changes that accompany the shifts in motility induced by GMFG loss. These are points for future examination which could provide new insights as to the regulation of lymphocyte adhesion.

## Conclusions

GMFG is required for lymphocyte migration. The loss of GMFG results in the selective increase of integrin expression, leading to altered uropod retraction and reduced persistence of leading edge protrusions. These changes could be a result of either the direct actions of GMFG on integrin properties or an indirect consequence of possible alterations in cytoskeletal dynamics associated with GMFG loss. It is unclear if the effects of GMFG loss on chemotaxis derive from the inability of cells lacking this protein to respond to chemotactic stimuli or their failure to sustain the directionality required for chemotaxis. Collectively this data suggests that GMFG plays an essential role in the regulation of lymphocyte movement.

## Methods

### Materials

Unless otherwise noted, all reagents and materials were obtained from Invitrogen. Transwell inserts were obtained from Corning Inc. Life Sciences (Lowell, MA, USA). The GMFG antibody (13625-1-AP) was obtained from Proteintech (Chicago, IL, USA), the ACTR2 antibody (ab49674) was obtained from Abcam (Cambridge, MA, USA) and the ARPC2 (07–227) and HUTS-4 (MAB2079Z) antibodies were obtained from Millipore. Integrin subunit antibodies were prepared in-house. The antibody to CXCR4 (MAB170), recombinant SDF1 and recombinant IL-2 were purchased from R&D Systems (Minneapolis, MN, USA). Integrin antibodies to α5 (JBS5), β1 (JB1A and 3S3), and LFA-1 were prepared in-house. The protease inhibitor cocktail (P8340) was obtained from Sigma. Fibronectin was obtained from Chemicon.

### Cell lines and tissue culture

All cell lines were maintained at 37°C in a humidified 5% CO_2_ atmosphere. The human lymphoid cell lines, Jurkat and CCRF-CEM, were cultured in RPMI 1640 medium supplemented with 10% FBS. Cells transfected with shRNAmir constructs were maintained under standard conditions with the addition of 1 μg/mL puromycin to the medium for selection.

Peripheral blood mononuclear cells (PBMC) were isolated from the whole blood of healthy donors by Ficoll gradient centrifugation. The cells were washed twice with PBS, resuspended in AIM V CTS Serum Free Medium (Invitrogen) and stimulated for 16 hours with phytohaemagglutinin P (5 μg/mL). The nonadherent lymphocytes were harvested by centrifugation (500 × g for 5 minutes) and resuspended in fresh medium supplemented with IL-2 12.5 ng/mL. All cell lines were subcultured in fresh IL-2 containing media every 2–3 days. All samples were obtained with informed consent using a protocol approved by the University of Manitoba, Research Ethics Board.

### Cell migration assays and pseudopodia isolation

Migration assays were performed as a modification of a previously described method [20], and the same protocol was employed for all cell lines. Prior to performing migration assays cells were split to a density of 2 × 10^5^ cells/mL and cultured overnight. Cells were collected by centrifugation (400 × g for 5 minutes), washed twice with serum free RPMI containing 0.1% BSA and resuspended in this medium prior to performing the assay.

Migration assays were performed in triplicate in 96 well plates containing inserts with 5 μm diameter pores according to the manufacturer’s instructions (Corning Life Sciences, Lowell, MA, USA). Briefly, 3 × 10^5^ cells were added to the upper chamber of each transwell and SDF-1 (50 ng/mL in RPMI + 0.1% BSA) was dispensed into the lower chamber of the transwells as chemoattractant. Cells were allowed to migrate for 2.5 hours at 37°C. Upon completion of the assay, cells were recovered from both the reservoirs and transwells and transferred to separate standard 96 well plates. Cell numbers were determined by the addition of 10 μL of Celltiter 96 Aqueous One cell proliferation assay reagent (Promega Corp.) to each well followed by incubation at 37°C for 3 hours. Percent migration was calculated from the ratio of cells in the reservoir to the initial input (sum of reservoir plus transwell), and multiplying by 100. Spontaneous migration was consistently measured to be less than 5% of the total cell number and was discounted for the purposes of this calculation.

### Pseudopodia isolation and analysis

Jurkat cells (7 × 10^6^/24 mm 3.0 μm diameter pores transwell insert) were stimulated with SDF1 (50 ng/mL) for 90 minutes and the pseudopodia on the underside of the insert were solubilized briefly (~1 second) in a 200 μL droplet of solubilisation buffer (10 mM ammonium bicarbonate, pH 7.4/1.0% SDS/2 mM sodium vanadate/protease inhibitor cocktail). Control whole cell samples were prepared by lysing 5 × 10^6^ Jurkat cells in the same solubilisation buffer. Three biological replicates were prepared for both the pseudopodia and control samples. Samples were stored at −20°C until analysis.

LC-MS/MS was performed as previously described (Dwivedi et al., 2008) using a nanoflow Tempo LC system (Eksigent, Dublin, CA) coupled to a Qstar Elite mass spectrometer (Applied Biosystems, Foster City, CA). Each digest was resuspended in 50 μL of 2% acetonitrile/1% formic acid in water (v/v). A 20 μL volume of each digest was analysed and each fraction was injected twice to provide technical replication of the LC-MS/MS analysis.

### Mass spectrometry data analysis

The raw MS data files were converted to the Mascot generic file format using the Mascot.dll script included with Analyst QS. The files corresponding to the fractions of individual samples were concatenated and submitted for protein identification using the X!Tandem search engine that is a part of the Global Proteome Machine. Standard settings were used for the analysis of QTOF data. Searches were performed against the human sequence database. Carbamidomethylation of cysteine was selected as a consistent modification, while oxidation of methionine and tryptophan as well as deamidation of asparagine and glutamine were selected as potential modifications. The measurement error window was set to 100 ppm and trypsin was selected as the protein cleavage specification.

### Virus packaging of shRNAmir constructs

Virus packaging was accomplished according to the methods provided by the manufacturer (Thermo Scientific Open Biosystems Expression Arrest – The RNAi Consortium (TRC) Lentiviral shRNA, Technical Manual, Open Biosystems, Thermo Scientific Inc.).

### Transduction of T cells and western blot assessment of knockdown

Cells (3 × 10^5^) in 0.5 mL of RPMI 1640 with 10% FCS were mixed with 0.5 mL virus containing supernatant at an MOI of 1 and incubated at 37°C for 2 hours. The cells were spun at 200 × g for 10 minutes and resuspended in 1 mL of RPMI 1640 with 10% FCS for culture. Transduced cells were positively selected by addition of puromycin (1 μg/mL) to the media after 72 hours of culture. The efficiency of silencing of target protein expression was assessed by western blot. Aliquots of transduced cells were collected and lysed on ice for 2 hours in lysis buffer containing 50 mM Tris (pH 7.5), 1% SDS, protease inhibitor cocktail (Sigma-Aldrich, St. Louis, MO, USA), and 0.1% benzonase nuclease (v/v of E1014-25KU, Sigma-Aldrich, St. Louis, MO, USA). The samples were centrifuged at 10,000 rpm for 5 min and 20 μg total protein from each sample was separated on a 4–10% SDS-PAGE. The proteins were transferred to nitrocellulose membrane and blotted with appropriate primary antibody and HRP-conjugated secondary antibody. Blots were developed by enhanced chemiluminescence (ECL). A blot of GAPDH was used to obtain a measure of sample loading and the GAPDH signal was used to normalize target specific bands among samples. For PBMC, knockdown was estimated using immunofluorescence microscopy and is described below.

### Immunofluorescence microscopy

96-well glass bottom plates were coated with fibronectin by incubating for 1 hour at room temperature or overnight at 4°C with a solution of 10 μg/mL fibronectin in sterile saline. Wells were rinsed three times for 5 minutes with sterile saline to remove any residual soluble fibronectin. T-lymphocytes were diluted to a density of 5 × 10^5^ cells/mL in Hank’s Buffered Salt Solution (HBSS) + 0.1% BSA and 100 μL of cell suspension was dispensed into individual wells. SDF1 was added to a final concentration of 50 ng/mL to induce random migration and polarization. Cells were incubated for 20 minutes at 37°C, after which the cells were fixed by the addition of 50 μL of 4% paraformaldehyde (v/v) in saline for 10 minutes at 37°C. Care was taken to maintain the temperature of the plate before and during this step. After carefully aspirating the fixing solution from each well, fixation was quenched by the application of 100 μL of 10 mM ethanolamine in PBS for 5 minutes at room temperature. Cells were permeabilized by treatment with 0.1% (v/v) Triton X-100 for 10 minutes at room temperature followed by a single wash with saline.

Primary antibodies were applied at 1 μg/mL in saline and incubated for one hour at 37°C. Cells were washed three times with saline prior to the application of secondary antibodies (Goat anti-Rabbit IgG-Cy2, GoatαMouse IgG-AlexaFluor488, or Goat anti-Rabbit-AlexaFluor594) at a dilution of 1:200 in saline. Cells were incubated with the secondary antibodies for 30 minutes at 37°C followed by a single wash with saline. 100 μL of 3% n-propylgallate in 90% glycerol was added to each well as an antifade reagent. Cells were imaged immediately following staining on a Zeiss Observer. Z1 microscope using a Zeiss Plan-Apochromat 63x/1.4 or 40x/0.95 objective. Confocal imaging was performed on a Zeiss LSM 710 Observer station using the same objective. Image acquisition and processing was performed using the AxioVision v4.8.0 software used to operate the microscope.

### Flow cytometry

Cells (1 × 10^6^ cells/sample) were prepared for flow cytometry analysis by fixation in 1% paraformaldehyde for 10 minutes, followed by a 10 min quench in 10 mM ethanolamine. Fixed cells were incubated with the appropriate primary antibody prepared as a 1:200 dilution in PBS for 1 hour at room temperature. Cells were then washed twice with PBS and incubated for 30 min with an AlexaFluor 488 conjugated goat anti-mouse secondary antibody. Samples and appropriate staining controls were analysed using a custom built FACSArray (BD Biosciences) fitted with a 96-well plate sample delivery system and blue sample excitation laser. Data analysis and figures were prepared using FlowJo software (version 7.5.5).

### Adhesion assays

96 well plates were coated with fibronectin (10 μg/mL in PBS) or ICAM-1 (5 μg/mL in PBS) for one hour at room temperature. Cells were initially labeled with the fluorescent reagent Calcein-AM (Invitrogen) according to the manufacturer’s instructions. Washed cells were plated at 2.5 × 10^4^ per well in 100 μL of media and incubated at 37 C for 30 min. Adhesion was assessed by comparing the levels of adherent cells following aspiration as a percentage of the total cellular input. The number of cells retained was estimated based on fluorescence readings taken from each well at the end of the assay. The results were then expressed as the percent adhesion for non-silencing control cells (which was taken as 100% of ‘normal’ adhesion) to enable simplified comparison of replicate experiments and different cell types. In assays employing the RGD peptide as an inhibitor of integrin binding, RGD peptide was added at a concentration of 250 μg/mL to the cells prior to plating them on fibronectin.

### Statistical analysis

For migration and adhesion assays, samples and controls were measured in triplicate. The standard error of the mean was calculated and used to indicate error ranges all graphs. Probabilities of statistical significance were calculated using a student’s *t*-test, and p-values were reported on graphs and in figure legends. All calculations were performed in Sigmaplot (v11).

## Authors’ contributions

DNDL performed all experimental work, participated in the design of the studies and contributed to the writing of the final manuscript. JAW conceived and designed the original studies and he co wrote the manuscript. Both authors read and approved the final manuscript.

## Supplementary Material

Additional file 1 **Microsoft Excel table. **List of peptides identified by mass spectrometry from GMFG. This table includes a list of peptides from the protein GMFG that were identified as part of a larger proteomics screen of T cell pseudopodia. Click here for file

Additional file 2 **Windows media player video clip.** GMFG-KD cell movement video #1. This video clip shows an example of the movement of a GMFG-KD primary T lymphocyte on a fibronectin coated surface. Click here for file

Additional file 3 **Windows media player video clip.** GMFG-KD cell movement video #2. This video clip shows an example of the movement of a GMFG-KD primary T lymphocyte on a fibronectin coated surface. Click here for file

Additional file 4 **Windows media player video clip.** GMFG-KD cell movement video #3. This video clip shows an example of the movement of a GMFG-KD primary T lymphocyte on a fibronectin coated surface. Click here for file

Additional file 5 **Windows media player video clip.** GMFG-KD cell movement video #4. This video clip shows an example of the movement of a GMFG-KD primary T lymphocyte on a fibronectin coated surface. Click here for file

Additional file 6 **Windows media player video clip.** Control cell movement video. This video clip shows an example of the movement of a control primary T lymphocyte on a fibronectin coated surface. Click here for file
